# Bayesian mixture model for accurate assessment of monthly maximum wind speed: A case study in Gwadar

**DOI:** 10.1016/j.heliyon.2024.e39383

**Published:** 2024-10-18

**Authors:** Tasir Khan, Yejuan Wang

**Affiliations:** School of Mathematics and Statistics, Gansu Key Laboratory of Applied Mathematics and Complex Systems, Lanzhou University, Lanzhou, 730000, PR China

**Keywords:** Comparison wind speed distribution, Mixture of sine-skewed-von mises distributions, MLE, Heterogeneous distribution, Gwadar

## Abstract

Assessment of monthly maximum wind speed (MMWS) is essential in various environmental disciplines, including architectural risk assessment, climatology, renewable energy sources, building design, and agricultural activities. The distribution of wind speed is an important module of its development. Presently, several mixing distributions have been suggested for fitting the theoretical maximum wind speed distributions. However, the drawback of various distribution elements is the outcome of numerous fitting presentations, and it is challenging to choose the best form of single distribution to employ when building a hybrid model. In order to solve those difficulties, utilizing the mixtures of Sine-skewed-von Mises distributions (MSS-VM) with various prior parameter distributions is extended in the present study. In this research, the assessment of MMWS employing the maximum-likelihood estimation (MLE) method is taken into consideration for the wind speed data from the Pakistan province of Baluchistan Gwadar station. The results of the Anderson Darling (AD), Chi-square, BIC, and AIC tests, as well as the Kolmogorov Smirnov (KS) test, indicated that MSS-VM was the best distribution for the Gwadar station. The suggested models automatically find the optimum number of elements, in addition to having more appropriate performance. The outcomes demonstrate that MSS-VM performance is more suitable than other heterogeneous and single-distribution models. The design estimations derived using the Bayesian mixture distribution provide a sense of the highest wind speed that will occur across a particular area. It is therefore crucial for designers and policymakers in the planning, designing, and building of various structures.

## 1 Introduction

The wind represents a highly influential, naturally occurring phenomenon of the environment that affects a wide range of other variables. For instance, the topography that winds blow over affects both wind speed and temperature, which consequently causes other harmful climatic events like dust storms, typhoons, and hurricanes [1]. Currently, the single distribution most widely used and recognized parametric approach to describing wind speed is the distribution of Weibull [[2], [3], [4]]. Additional widely used distributions, like the Gamma [5], and Lognormal [6], are also taken into account when fitting distributions of the wind speed. The authors confirmed that the generalized Gamma distributions can fit the wind speed distributions with reliability [7]. According to Ref. [8], most wind speed data in the UAE can be more accurately fitted by the Log Pearson III, Generalized-Gamma, and Kappa distributions, according to the study on the L-moment ratio diagram. A five-parameter Wakeby probability distribution was found to be an appropriate fit for the information on wind speeds in Germany [9]. The characteristics of additionally typical distributions, such as inverse Burr, Marshall-Olkin Power Lomax [10], generalized extreme value (GEV) [11], and inverse Gamma [12] distributions, were also investigated in several areas.

### 1.1 Heterogeneous mixture distributions

In [13] using normal, extreme-value type 1, Weibull, and Gamma distributions, twelve heterogeneous and homogeneous mixed distributions are generated, and they are utilized for fitting the average data on wind speeds in the United Arab Emirates. Results showed that out of all heterogeneous and homogeneous mixture distributions, as well as single models, the Weibull Extreme Value type-one mixture distribution achieved better than others. A singularly truncated normal Weibull mixed probability (TN-W) was proposed in Ref. [14] to study wind speed features of Elazig Keban, Elazig, and Elazig Madden sites. TNW demonstrated superior distribution fit abilities when contrasted with two-component Weibull mixture distribution (WW). Based on the results presented in Ref. [15], the mixture Ga-W distribution is superior to the N-W, Weibull-Weibull, and normal mixture distributions in terms of estimating wind speed distribution. An algorithm built using Burr-GE, Wakeby, and Kappa suggested a method for estimating wind distributions worldwide [16]. The authors of [17] demonstrated that, out of ten mixing distributions, W-LN worked particularly well in Aljouf, whereas Gamma-Rayleigh performed best in Sharurah. In Ref. [18], some mixed heterogeneous distributions were suggested to generalize extreme values (Lognormal, Weibull-Lognormal, and Weibull-GEV). The related results showed that GEV-LN performed well in fitting the popular bell-shaped unidirectional distribution, but W-GEV was able to fit both bi-modal and unimodal wind distributions effectively. The fit performance of 23 mixtures and 20 single models for the wind distribution was investigated in Ref. [19]. The findings demonstrated that mixture distributions outperformed single distributions in most cases, including Dagum-GEV, Burr-GEV, GEV-W, and Dagum-Weibull.

As the influence of single distributions is limited many other researchers are creating mixed distributions. The results reported in the latest literature indicate that mixture models perform better than single models, especially in complex wind regimes with several modalities. Bimodality is a key focus of this study. Therefore, in this research, we also emphasize the use of mixture distributions to model wind speeds in various wind regimes. Nevertheless, there are constraints in achieving improved fitting performance through mixture modeling. The most challenging of the various distribution models to take into consideration while building the mixture distribution is that, according to recent literature, users make a subjective decision about the number; the number is chosen to be two in most heterogeneous mixture models [13,19,20]. Ouarda et al. [8], Fawad et al. [21], and Goel et al. [22] emphasized the three basic assumptions of stationarity, independence, and homogeneity, which are thought to be important when performing wind speed analysis. If such assumptions are violated, structural design estimations may be misrepresented, which may limit their application.

The Bayesian technique for symmetric directions especially, Bayesian analysis utilizing the symmetric von Mises-Fisher and von Mises distributions, has been widely studied in the literature [[23], [24], [25], [26]]. The mixture model von Mises Fisher is employed by Roge et al. [27] and Taghia et al. [28] and Mulder et al. [29] emphasize non-informative priors while providing a Bayesian model of inference for von-Mises mixture distributions utilizing the reverse run Monte Carlo Markov chain (MCMC) generator. It is evident from the previous that there exists a gap in the scientific literature, which motivated us to suggest a novel application of Bayesian estimation for wind data. Skew Wrapped Cauchy mixing model and skew von Mises-Fisher distribution have been exposed to Bayesian estimation [30,31]. The research gap related to the inadequate comprehension and examination of wind speed distributions, specifically with the application of mixture distributions such as the sine-skewed-von Mises (SSVM) distribution model in wind energy evaluations. Although much research has concentrated on single distribution models, like the Rayleigh, Weibull distributions and several others [[32], [33], [34], [35]]. There aren't many thorough examinations of how effectively mixture distribution models describe wind speed data under different circumstances and at different elevations.

The study proposes a novel mixture of the sine-skewed von Mises (MSS-VM) distribution, which enhances the modeling of wind speed data and multimodal behavior that are often observed in wind speed distributions. The research utilizes Bayesian estimation methods to derive a posterior distribution for estimating monthly maximum wind speeds. This approach not only improves the accuracy of the parameter estimates but also provides a robust framework for quantifying uncertainty in wind speed predictions. The effectiveness of the proposed Bayesian method, the study conducts a simulation analysis. This empirical evaluation demonstrates how well the MSS-VM model performs in estimating wind speed distributions. The study includes a thorough comparison of the proposed MSS-VM model with several other mixture distributions and established single distributions.

The remaining portion of this work is structured as follows: Study area is discussed in Section 2, material and methodology are discussed in section 3, and results are presented in section 4, and Section 5 presents a conclusion and recommendation.

## 2 Study area and data

A nation's economic growth and development are significantly influenced by its energy sector. Renewable sources of energy such as solar and wind power are accessible and affordable. Pakistan has not adequately investigated the possibilities of using alternative technology. Pakistan's energy consumption has increased dramatically over the last few decades and is predicted to continue rising. The primary energy resources of Pakistan include nuclear, hydro, natural gas, and oil. Pakistan has experienced a serious crisis of electricity in recent decades, which has resulted in poor economic growth because of insufficient energy strategy [36,37].

**Fig. 1:**
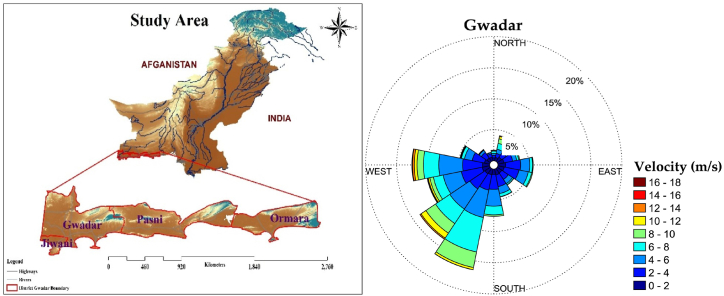
Study area and Present direction of wind speed in district Gwadar.

The Meteorological Department of Pakistan provided data on monthly maximum wind speeds from 1981 to 2019. Table 1 shows the statistical indicators, including mean, standard deviation, coefficient of variation, minimum, maximum, skewness, and kurtosis. The Gwadar district of Baluchistan meteorological station is situated at an elevation of 29.86 m, 25° 08′ latitude, and 62° 20′ longitude. At 10-min intervals, maximum wind data is recorded, which includes wind speed in meters per second at heights of ten- and 50-m. Typical wind speeds of higher than 9.2 miles/h, the windier portion of the year covers 6.1 months, from 3 March – 6 September. In Gwadar, May is the windiest month of the entire year, with an ordinary hourly wind speed of 10.4 mph [38,39]. Fig. 1 shows the geographical positions and displays in the Gwadar station wind speed directions. Fig. 1 makes it very clear that the station's wind velocity direction is looking southwest.

**Table 1:** Presents the descriptive statistics of Gwadar station.

Gwadar	Mean	S D	CV	Min	Max	Skewness	kurtosis	latitude	longitude	Elevation
10m	6.368	0.715	11.23	4.03	8.63	−0.15	0.20	25° 08′	62° 20′	29.86
50m	7.505	0.721	9.62	5.49	10.08	0.37	0.42	25° 08′	62° 20′	29.86

Gwadar has an arid environment that is hot and dry, and it is situated between 0 and 984 ft above sea level. The effect of the ocean keeps the temperature higher in the winter and lower in the summer. June is the hottest month with an average temperature that stays between 31 °C and 32 °C. January's coldest month with an average temperature ranging from 18 °C (64 °F) to 19 °C (66 °F). One distinctive feature of the Balochistan coastline region is its consistent temperature [40]. The winters are comfortable, except occasional cold spells brought on by winds sweeping down the Balochistan desert. Winters in Gwadar are shorter than summers [41]. The climate in Gwadar is the same as that of the Middle East because the majority of the rain falls between December and January. On June 6, 2010, the most rainfall previously recorded 227 mm or 8.9 inches in 24 h was recorded.

Gwadar station was selected for this study due to its unique coastal location along the Arabian Sea. This makes it ideal for capturing wind patterns influenced by both land-sea interactions and regional climatic phenomena, such as the monsoon. Additionally, Gwadar's proximity to important maritime routes and its development as a port city further highlight the significance of understanding local wind dynamics, which can impact shipping, infrastructure, and renewable energy projects, particularly wind energy potential.

## 3 Materials and methods

### 3.1 Candidate probability distributions (PDs)

In the present study, Wind speed analysis was calculated using many distributions. These distributions, which are frequently employed in the Analysis of exceptional occurrences, included five single distributions, and multiparameter mixture distributions. These are Gamma (GAM), Generalized Gamma (GG), Dagam3, Fatigue Life (3), Inverse Gaussian (IGA), Weibel (3), Beta, (NAK), Jonson Su (JON), Normal (N), Kumaraswami, Log gamma (LG), Kumaraswamy-normal, Nakagami-Gumbel, Dagum-Beta, Log normal-log Pearson, Log logistics-inverse gaussian, Normal-Gamma distributions. The majority of such distributions were proposed for on-site wind speed and analysis across multiple countries [[42], [43], [44], [45], [46], [47], [48], [49], [50]].

### 3.2 Estimation parameters of PDs by the maximum likelihood estimation

The MLE approach was utilized in this study to estimate the characteristics of PDs. It was previously applied in the frequency estimation of wind speed [[51], [52], [53], [54]]. The MLE approach is typically employed when estimating a population parameter from sample data on wind speed. RA Fisher established and promoted this technique between 1912 and 1922 [55]. Estimation of parameters is used in the assessment of the method of a linear method to produce the maximum. Probability that an observation will be made. The probabilistic function can be defined as a collection of values for the parameters, for distribution with a probability density function (pdf) provided by f(x) and PDF from the Union of Observing Condition [56].

### 3.3 The sine-skewed von mises distribution

The probability distribution is called the *sine-skewed von-Mises distribution*. This model is an extension of the classical von-Mises distribution, which is widely used for modeling circular data (data that represents angles or directions) [57]. The von-Mises distribution is often referred to as the circular analog of the normal distribution due to its symmetrical, unimodal nature. However, the classical von-Mises distribution is limited in its ability to capture asymmetry or skewness. To address this limitation, we introduce skewness into the von-Mises distribution using a sine function, resulting in the sine-skewed von-Mises distribution [58]. This modification allows the distribution to model both symmetrical and asymmetrical circular data. Nature, making it more flexible in capturing real-world phenomena that exhibit directional bias or asymmetry [59].

Numerous distributions on a single circle possess a common feature: they are symmetrical regarding their position μ ∈ [−π, π]. But, since the symmetry assumption in data is frequently rejected [60], introduces the k sine skewed von Mises distribution and density function as follows:

[1] $$f_{SSVM}\left(\theta ;\mu ,\tau ,\lambda \right)=\frac{1}{2\pi I_{0}\left(\tau \right)}\exp\left[\tau \;\cos\left(\theta -\mu \right)\right]\left[1+\lambda \;\sin\left\{k\left(\theta -\mu \right)\right\}\right]$$

Here $I_{0}\left(.\right)$ is the initial type of level 0, a location factor, μ ∈ [−π, π), τ > 0 is the parameter of concentration, the measure of skewness is −1<λ≤1, and a positive integer is k. The distributions are skewed left when λ > 0, and the distributions are skewed right when λ < 0. The expression for a combination of SSVM distributions by $M\in Z^{+}$ elements are

[2] $$f_{M}\left(\theta ;w,\mu ,\tau ,\lambda \right)=\sum_{i=1}^{P}w_{i}f_{SSVM}\left(\theta ;\;\mu_{i},\tau_{i},\lambda_{i}\right)$$

Where the parameter vectors are $\mu =\left(\mu_{1},\mu_{2},\ldots ,\mu_{p}\right)\text{,}$ $\tau =\left(\tau_{1},\tau_{2},\ldots ,\tau_{p}\right)\text{,}$ and $\lambda =\left(\lambda_{1},\lambda_{2},\ldots ,\lambda_{p}\right)\text{,}$ $\lambda_{i}\in \left[-1,1\right]\text{,}$ $\mu_{i}\in (-\pi .\pi$) and $\tau_{i}>0$*,* Weights that represent the relative proportions of every element in the entire sample which is represented by the vector $w=\left(w_{1},w_{2},\ldots ,w_{p}\right)$, $\sum_{i=1}^{P}w_{i}=1$. Algorithm1 was implied to create a sample based on the SSVM distribution equation (1). The proof of Sine-Skewed von Mises Distribution is discussed in Appendix B.

Algorithm 1 processes to create a sample based on the SSVM.

1 Create $\Theta_{VM}$ base on Von-Mises distribution given parameters *k* and μ.
2 Calculate *U* using *U* (0.1).
3When U<(1+λsin(Θ−μ)/2, as a result $\Theta_{SSVM\;}=\Theta_{VM}$.

### 3.4 Estimation parameters of PDs by the maximum likelihood estimation

The MLE approach was utilized in this study to estimate the characteristics of PDs. It was previously applied in the frequency estimation of wind speed [[51], [52], [53], [54]]. The MLE approach is typically employed when estimating a population parameter from sample data on wind speed. RA Fisher established and promoted this technique between 1912 and 1922 [55]. Estimation of parameters are used in the assessment of the method of linear method to produce the maximum. Probability that an observation will be made. The probabilistic function can be defined as a collection of values for the parameters for distribution with a probability density function (pdf) provided by f(x) and PDF from the Union of Observing Condition [56].

The mixture of sine skewed Von-Mises in equation (2) can be represented by the function of log-likelihood as follows:

[3] $$l\left(w,\mu ,\tau ,\lambda /\theta \right)=\sum_{h=1}^{r}\log\left(\sum_{i=1}^{P}w_{i}f_{SSVM}(\theta ;\;\mu_{i},\tau_{i},\lambda_{i}\right))$$

The partial derivations of equation (3) about (w,μ,τ,λ), the MLE parameters (w,μ,τ,λ) could be acquired. Because there are no closed-form equations available, estimations should be obtained numerically. The MLE is calculated employing the Differential Evolution (DE) technique [61], which uses the DEoptim package in the R programming language [62]. DE is a heuristic evolution approach for universal optimization that is being extensively investigated for its substantial efficiency as a worldwide optimization method for continuously minimizing numerical problems. It is effective in many scientific and technology-related applications.

#### 3.4.1 Prior selection

The first stage in Bayesian analysis is to create a prior distribution, which captures the researcher's assumptions or understanding of the parameters before data observation. The choice of prior can significantly influence the results, especially when the sample size is small. Priors can be informative, reflecting strong prior beliefs based on previous studies or expert knowledge, or non-informative, aiming to exert minimal influence on the posterior distribution. In this study, the prior distribution for the parameters of the sine-skewed von Mises mixture model should be selected based on the predictable performance of wind speeds.

#### 3.4.2 MCMC settings

The posterior distribution is computed using MCMC techniques like Gibb's sampling and the Metropolis-Hastings algorithm, particularly in cases when the posterior distribution cannot be computed analytically. These procedures create samples from the posterior distribution by creating a Markov chain that has the desired posterior distribution as its symmetry distribution.

Some important MCMC settings are:

• A lot of beginning samples are eliminated in order to let the chain settle into a stationary distribution.
• Decreasing autocorrelation by keeping only the k kth sample in the chain
• The entire number of samples drawn, which must be sufficient to guarantee that the posterior distribution of the sample is well approximated.
• Techniques like trace graphs and the Gelman-Rubin statistic to determine if the MCMC method has converged.

### 3.5 Goodness of fit tests

The process of assessing probability distribution (PD), the technique used for parameter estimation, and the accessibility of information on wind speeds are some of the variables that affect the final selection of PD. In the current study, the Akaike information criteria (AIC), Bayesian information criteria (BIC), Anderson Darling (AD) test, and Kolmogorov-Smirnov (KS) criterion were employed to assess the sufficiency of various PDs. To analyze the wind speed, the previous graphic tests and goodness-of-fit tests were performed [13,[63], [64], [65], [66], [67], [68]]. The previously mentioned goodness of fit tests can be applied with confidence to select the most suitable distribution in environmental statistics.

#### 3.5.1 Kolmogorov-Smirnov (KS) test

It can be determined using the KS test; it depends on an empirical distribution function if the data set is taken according to a suggested distribution [65]. Assuming that we have a sample selected at random $\left(x_{1},x_{2},\ldots ,x_{n}\right)$ from the distributions, the EDF can be expressed as follows:

[4] $$F_{n}\left(x\right)=\frac{1}{n}\left[number\;of\;observation\leq x\right]$$

The highest vertical separation between the theoretical PD and EDF determines the KS statistic (D). The KS test statistic (D) is.

[5] $${D=\max_{1\leq i\leq n}}_{,}\left[F\left(x_{i}\right)-\frac{i-1}{n},\frac{i}{n}-F\left(x_{i}\right)\right]$$

F represents the theoretically cumulative distribution's function, xi represents the ith level of the data, and n represents the sample size in equation (5).

#### 3.5.2 Anderson-Darling (AD) test

The fit between an actual distribution model and the estimated distribution model is evaluated using the AD test. The downward trend of the distribution, which becomes a crucial feature in simulating extreme occurrences, is given more weight by the AD test [69]. The formula for the AD statistic A^2^ is as follows:

[6] $$A^{2}=-n-\frac{1}{n}\sum_{i=1}^{n}\left(2i-1\right)\left[\ln\;F\left(X_{i}\right)+\ln\left(1-F\left(X_{n-i+1}\right)\right)\right]$$

Where X represents the parameter being studied, $F\left(X_{i}\right)$ represents the distribution's performance, and n represents the size of the sample. A^2^ represents the test outcome in this particular case.

#### 3.5.3 Chi-Squared ($\chi^{2})$ test

The goodness of fit test in chi-square is employed to evaluate the relationship between the two sets of data. This equation can be used to calculate it:

[7] $$\chi^{2}=\sum_{i=1}^{r}\frac{{\left(o_{i}-N_{pi}\right)}^{2}}{E_{i}}=\sum_{i=1}^{r}\frac{{\left(o_{i}-E_{i}\right)}^{2}}{E_{i}}$$

where the predicted value represents $E_{i}$ and the actual value represents $O_{i}$. A significant level of relationship between the two sample distributions can be seen in the small $\chi^{2}$ test value.

#### 3.5.4 Akaike and Bayesian information criteria

The alternative approach, which includes both BIC and AIC, depends upon the relative measurement loss of material when using the model to characterize the observations [70]. Both of these methods, though, represent one of the most widely used techniques. The usual approach to AIC and BIC as

[8] AIC=−2log(L)+2K

[9] BIC=−2log(L)+Llog(n)

where L represents the log-likelihood function, *K* represents the parameter, and n represents the sample size. The model with the smallest values of AIC and BIC for the wind speed information has been selected as having the most optimal fit distribution.

## 4 Results

It is important to confirm the primary principle, namely the homogeneity, stationarity, and independence of the maximum monthly wind speed (MMWS), before doing deep learning analysis. Mann-Whitney test statistics are utilized to assess homogeneity, while L-Jung-Box Q (LBQ) test statistics are employed to assess stationarity and independence [71]. A non-parametric test was recommended by Mann and Whitney [72] to verify the homogeneity. This test recommends first dividing the initial sample into two samples of relatively similar length so that $n_{1}n_{2}$. The complete sample should now be arranged in ascending order, with each sample value being noted as belonging to either the first or second sample. The fundamental premise of this test is that if the ranking orders of the components in the first sample are consistently higher or lower than the ranking orders of another sample, the entire sample will not be homogeneous. The smallest value of “V" between $V_{1}$ and $V_{2}$ is what this test depends on, as seen below.

[10] $$V_{1}=n_{1}n_{2}+\frac{n_{1}\left(n_{1}+1\right)}{2}-R_{1}\text{and}\;V_{2}=n_{1}n_{2}-V_{1}$$

where R_1_ displays the first sample's overall ranking order as a sum. Under the null hypothesis, which states that the data set at present is homogeneous, Mann-Whitney claimed that the variance as well as the mean for both samples n_1_ and n_2_ are almost normally distributed with the mean and variance as follows:

[11] E(V)=n1n22,andVar(V)=n1n1(n1+n1+1)12

Also, the standard normal test statistic as

[12] $$U=\frac{V-E\left(V\right)}{\sqrt{Var\left(V\right)}}$$

The benefit of utilizing the LBQ test is that it is a composite test that examines randomness “overall” as opposed to testing randomness at each lag [73,74]. The lag duration should not be greater than ${\;n}_{1}/4$, in the LBQ test, where $n_{1}$ is the record length of the ith site, and the hypothesis to be set is that all K up to that lag is zero. In this test, H_0_: all the observed values are independent vs. H_a_: all the observed values are non-independent. The test statistic is

[13] $$LBQ=n\left(n+2\right)\sum_{k=1}^{m}\left[\frac{{\hat{\rho }}_{k}^{2}}{n-k}\right]∼\chi_{m}^{2}$$

In equation (13), m is the lag length, and ${\hat{\rho }}_{k}^{2}$ is the sample autocorrelation at lag k. At the selected level of significance, the null hypothesis is rejected if $LBQ>\chi_{1-\alpha ,m}^{2}$. It is the Box-Pierce Q statistic in its modified form. Both statistics have a chi-square distribution with m degrees of freedom when n is large. Comparing LBQ statistics to Box-Pierce Q statistics, the former is superior for both small and large sample sizes. Table 2 reveals that the LBQ statistic for each lag of the wind speed data series is insignificant (individually 10m and 50m), which is adequate proof that the fundamental series are stationary and independent. For the M − W test, every group has been split into two portions. The even number of observations series has been split into two equal portions, while the other series are separated into around equal portions. All sequences display insignificance outcomes since 10m and 50m of Gwadar site p values are higher than 0.05, and it is therefore determined that the two groups of MMWS series have the same distribution. All testing revealed that every series was suitable for further analysis Table 2. In the case that certain MWMS sequence assumptions are not met, then more complicated statistical techniques must be used to account for the relationship between the data as it is observed and how it develops over time.

**Table 2:** Outcomes of fundamental assumptions for the Gwadar wind farms.

Stations	LJ-BOX		MWU-test	
Gwadar	Statistics -value	P - Value	Statistics -value	P - Value
10m	2.346	0.531	1.68119	.09296
50m	0.632	0.654	0.09181	.92,828

### 4.1 Probability Distributions (PDs) selection

The statistical analysis of the wind speed information is a critical instrument to use for wind farms, wind generators, and other purposes like irrigation. To assess the potential for wind energy in a particular area, it is important to accurately determine the probability distribution of the wind. In the beginning, the study took into consideration twenty PDs, i.e., Gamma (GAM), Generalized Gamma (GG), Dagam3, Fl (3), Inverse Gaussian (IGA), Weibel (3), Beta, Nakagami (NAK), Jonson Su (JON), Normal (N), Kumaraswami, Log gamma (LG), KUM-N, NAK-GUM, JON-DAG, DAG-B, LN-LP, LL-IGA, N-GAM, and Mixture SSVM. The goodness of fit methods were employed to evaluate the PDs, including the chi-square test, Anderson Darling, Kolmogorov Smirnov, AIC, and BIC. The assessments based on R^2^, AIC, BIC, AD, and KS criteria for the most accurate fit distributions are listed in Table 3. According to the smallest goodness of fit tests amongst the twenty PDs taken into consideration during the research, the best-fitted distribution was selected for Gwadar stations at 10 m and 50 m. The most significant outcomes for every PD are shown in Table 3. Each of the models under consideration will be applied and used to estimate the maximum wind speed distributions on wind speed databases, taking into account the specifications of the various models. Five goodness of fit criteria are calculated for each of the models. Table 3 shows the results of multiple models at Gwadar station 10m. Among the twenty models, the mixture SSVM distribution model performed best concerning all goodness of fit indices except KS, AD, chi-square, R^2^, and BIC, and KUM-N is also the most effective wind speed distribution model for Gwadar 10m. Interestingly, the predicted distributions of wind speed are produced by a mixture of SSVM. Regarding the four goodness-of-fit measurements, Kumaraswamy outperforms each of the other approaches out of the twelve single-distribution models. The mixture of SSVM is superior to KUM-N when these two are compared. Generally, mixture SSVM distributions outperform singular distributions and heterogeneous mixture distributions in this set of data. In Gwadar 50m, as shown in Table 4, in terms of KS, AD, and BIC, MSSVM is the best model, while NAK-GUM performs the best in terms of AD, KS, BIC, AIC, and all heterogeneous mixture distributions (MSSVM) and outperforms the other models under comparison concerning the distribution fitting of wind speeds. Among single and heterogeneous mixture distributions, the optimal distribution models for Gwadar 50m are Log gamma, NAK-GUM, KUM-N, DAG-B, and MSSVM, respectively.

**Table 3:** Statistical assessment of different distribution functions for 10m Gwadar station.

Gwadar 10m	Models	KS	AD	*χ*2	AIC	BIC
Single	GA3	0.055	1.348	16.039	4750.93	4781.83
	GG	0.052	1.028	16.548	4751.04	4782.15
	DAG	0.070	2.458	20.013	4753.39	4784.21
	FL (3)	0.044	0.820	12.808	4740.81	4771.51
	IGA (3)	0.045	0.849	11.341	4738.07	4769.05
	WE (3)	0.037	0.707	7.911	4733.67	4764.56
	B	0.040	0.602	11.702	4739.03	4769.04
	NAK	0.052	1.163	16.947	4750.53	4782.45
	J SU	0.062	5.398	14.261	4745.91	4776.92
	N	0.046	0.806	13.181	4743.68	4774.73
	KUM	0.038	0.702	12.618	4734.70	4765.71
	L. GA	0.582	0.821	17.675	4744.21	4775.31
Heterogeneous	KUM-N	0.027	0.315	4.817	4727.03	4758.04
	NAK-GUM	0.031	0.359	7.998	4732.54	4763.43
	LP-GEV	0.038	0.948	6.637	4735.99	4766.92
	DAG-B	0.031	0.347	5.716	4729.99	4758.85
	LN-LP	0.035	0.592	11.40	4737.67	4769.57
	LL-IGA	0.036	0.464	9.443	4736.81	4767.71
	N-GAM	0.034	0.671	5.739	4734.90	4765.84
Mixture	MSS-VM	0.020	0.232	4.182	4724.67	4743.39

**Table 4:** Statistical assessment of different distribution functions for 50m Gwadar station.

Gwadar 50m	Models	KS	AD	*χ*2	AIC	BIC
Single	GA3	0.025	0.389	3.788	3753.93	3818.83
	GG	0.025	0.379	7.942	3760.04	3821.15
	DAG	0.024	0.377	4.262	3763.39	3812.21
	FL (3)	0.025	0.400	4.512	3761.81	3825.51
	IGA (3)	0.026	0.363	3.671	3758.07	3815.05
	WE (3)	0.025	0.395	3.925	3753.67	3822.56
	B	0.024	0.352	3.667	3750.03	3811.04
	NAK	0.026	0.421	8.076	3751.53	3827.45
	J SU	0.023	0.352	6.193	3745.91	3819.92
	N	0.028	0.424	5.971	3743.68	3814.73
	KUM	0.025	0.384	4.262	3734.70	3810.71
	L. GA	0.023	0.304	3.390	3754.21	3814.31
Heterogeneous	KUM-N	0.023	0.315	2.830	3755.03	3808.04
	NAK-GUM	0.020	0.266	4.794	3748.54	3805.43
	LP-GEV	0.023	0.316	6.629	3751.99	3813.92
	DAG-B	0.022	0.300	3.737	3759.99	3811.85
	LN-LP	0.024	0.351	3.668	3757.67	3808.57
	LL-IGA	0.023	0.331	3.813	3756.81	3807.71
	N-GAM	0.023	0.338	3.810	3754.90	3806.84
Mixture	MSS-VM	0.022	0.243	5.330	3743.67	3801.39

**Fig. 2:**
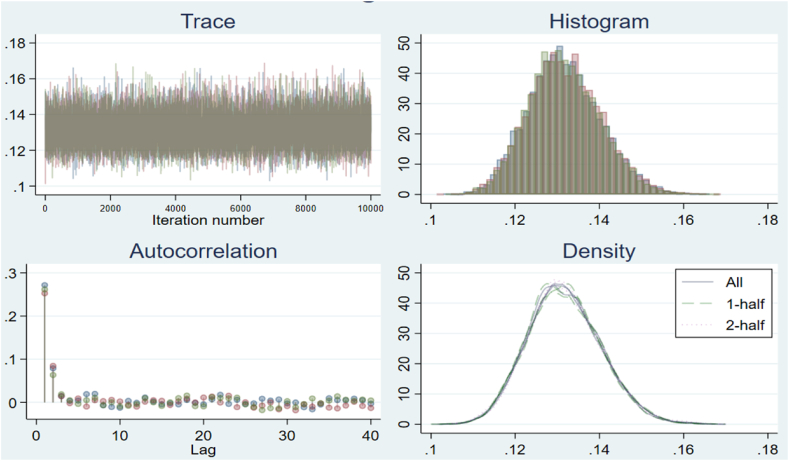
The trace, ACF, and density plots for Gwadar station 10m.

**Fig. .3:**
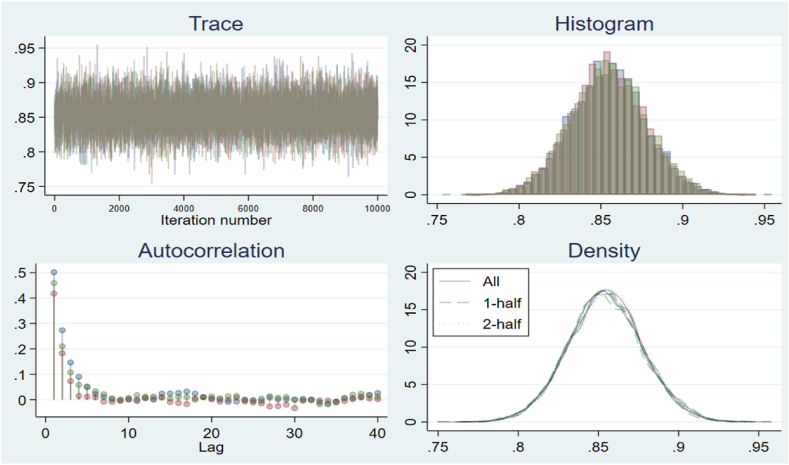
The trace, ACF, and density plots for Gwadar station 50m.

Fig. 2, Fig. .3 indicate the trace chain has been changed to a stationary distribution during long burns. The most noticeable aspect of stationary distributions from trace plots is a usually constant mean and variance. These figures similarly display a perfect trace plot. Because the chain's core looks to be near constant mean values with very minor changes. This suggests that the chain will reach the desired (right, stationary) distribution. Kernel density charts show that the Bayesian point estimate is the average posterior mean or median, and the range of 0.77th to 95.5th percentile is the 95 % Bayesian confidence interval, also known as the credible interval. The numerical outcomes of the variables produce a graphical representation with similar results, the MCMC output (trace, ACF, and density plots), produced by STATA software. As a result, the posterior kernel density is meant to be stable and convergent across all parameters. Auto-correlation function plots for each parameter chain also reveal the dimensions of the posterior distribution that are rapidly mixing. Rapid mixing is frequently linked with a low posterior correlation among metrics. The graphs show that all parameters mix well, with autocorrelation vanishing before five delays in each case.

**Fig. 4:**
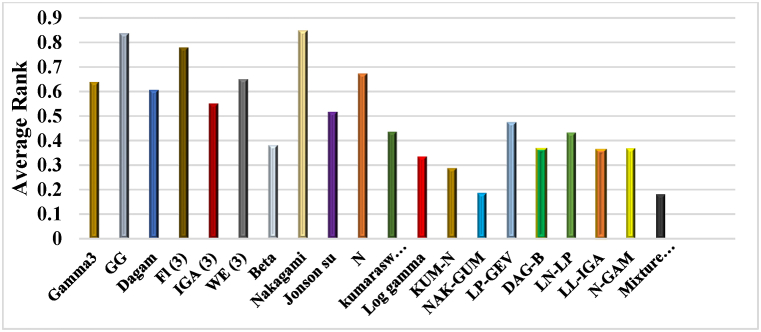
Twenty wind speed distribution models' average ranks on Gwadar databases.

Fig. 4 shows the average rankings for all models of distribution that have been computed. The conclusions are as follows:

• Single distributions like Beta and Log-Gamma will provide greater flexibility and ranking and outperform each of the single distributions using fewer parameters for modeling in terms of fitting.
• Average (mean) rankings of mixture heterogeneous distributions are more significant. Compared to the majority of single distributions, the former cases might benefit from various distributions' fitting capabilities when there are fewer elements within mixture distribution modeling.
• MSS-VM outperforms all other models. It also performs more suitably than other heterogeneous mixtures and single distribution models as well.

**Fig. 5:**
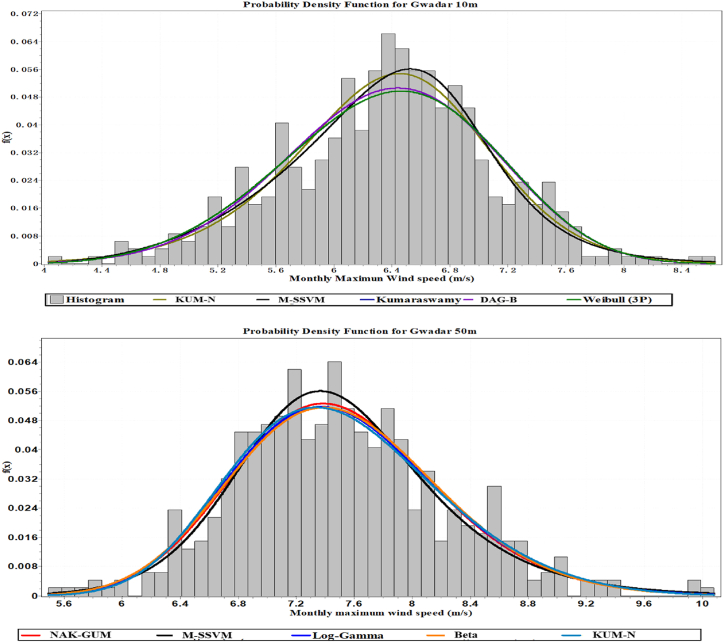
The Five most precise models identified wind speed probability density function distribution at Gwadar station.

**Fig. 6:**
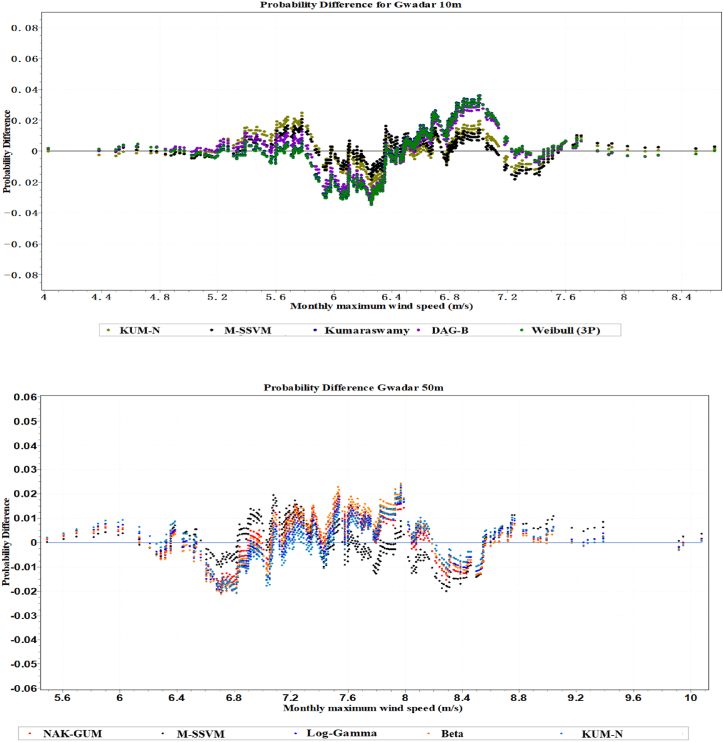
Probability difference of maximum wind speed for the five most accurate models at Gwadar station.

**Fig. 7:**
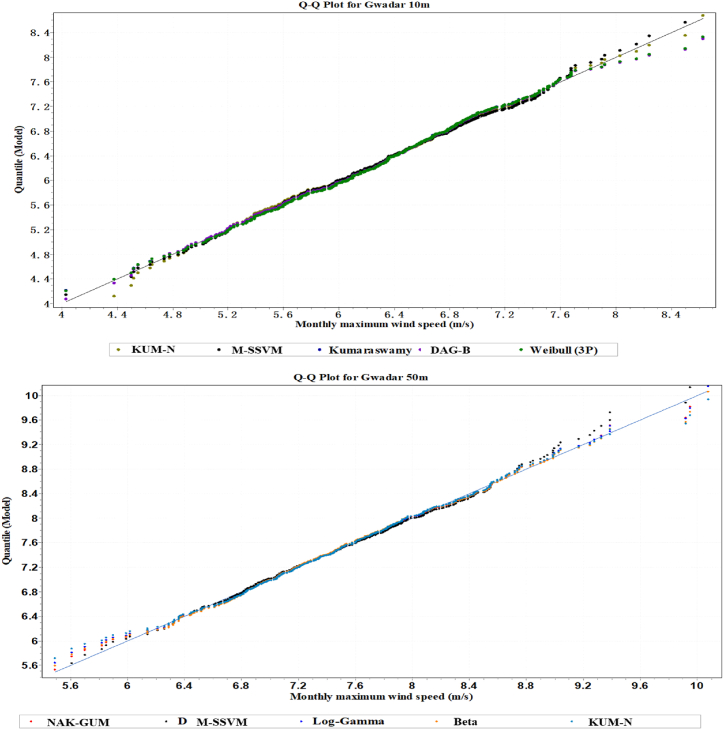
The quantile plots of maximum wind speed for the five most effective models at Gwadar station.

The estimation of the wind velocity distribution of the best five simulation models at the Gwadar site is displayed in Fig. 5. The two models that have been suggested, based on Fig. 5, have the capability of modeling empirically measured wind speed distribution in both unimodal and multimodal wind regimes. According to the probability difference and quantile plots of the top five models at 10m, they are M-SSVM, KUM-N, DAG-B, Kumaraswamy, and Weibel, and at 50m, they are MSS-VM, NAK-GAM, KUM-N, L.GA, and B respectively. The maximum wind speed distributions assessed for each of these models are displayed in Fig. 6. The wind velocity distribution for the best five simulation performance models at the Gwadar site is depicted in Fig. 7. Based on the findings from this figure, two models have been recommended for their ability to effectively model both unimodal and multimodal wind regimes using empirically measured wind speed data. At a height of 10 m, the top five models identified from the quantile plots include M-SSVM, KUM-N, DAG-B, Kumaraswamy, and Weibel. These models have demonstrated robust performance in capturing the distributional characteristics of wind speeds observed at this altitude. Similarly, at a height of 50 m, the recommended models based on their performance in modeling wind speed distributions are MSS-VM, NAK-GAM, KUM-N, L.GA, and B. These models are noted for their ability to handle the variability and complex patterns exhibited by wind speeds at higher altitudes in the Gwadar region. Overall, the analysis presented in Fig. 7 underscores the suitability of these selected models for accurately representing wind velocity distributions across different heights, providing valuable insights for wind energy assessments and related applications in the region.

The MCMC method discards 2500 first iterations. The burn-in period allows the algorithm to reach a stationary distribution, guaranteeing that the samples are pulled from the genuine posterior distribution rather than the initial values. The burn-in duration is chosen based on the specific problem and the MCMC chain's behavior. It is frequently determined by inspecting trace plots and autocorrelation plots to check that the chain has sufficiently converged before collecting samples for inference. Thinning is commonly done after the burn-in stage. For example, if the burn-in period is 2500 iterations the total number of iterations is 10,000, and you utilize 3 thinning, you would discard the first 2500 samples while keeping every third sample from the next 7500 iterations. The gamma distribution is used as a prior distribution for the estimating of parameters of the model.

The choice of Gwadar as a case study location is significant for several reasons, despite relying on data from a single measurement station. Gwadar, being a coastal city, is positioned at a key location where the interplay of land-sea interactions influences the wind patterns. Coastal regions are known for their distinct wind characteristics, which can vary from inland regions due to the proximity to large water bodies and the resulting temperature differentials. Gwadar experiences unique wind regimes, driven by both the local topography and regional climatic conditions. Its location at the junction of the Arabian Sea and the surrounding hilly terrain creates specific wind speed distributions, which may differ significantly from neighboring regions. A detailed case study of Gwadar can provide insights into the coastal wind characteristics, especially for areas with similar geographic features. Additionally, the selection of Gwadar aligns with the study's broader objectives of understanding wind energy potential in coastal regions, making this a representative choice. While data from a single station could be seen as a limitation, it is important to note that wind speed patterns tend to exhibit spatial coherence, especially in coastal regions where large-scale meteorological drivers dominate. Therefore, the station at Gwadar can serve as a reasonable representation of the local wind climate, allowing for meaningful conclusions about wind speed distributions. Future studies may include additional stations to further validate the findings.

Sensitivity analyses improve the comprehension of the reliability and durability of the MSS-VM model for predicting wind speed distributions. By examining parameter sensitivity, and input data variability, and conducting comparative assessments with other models, researchers can ensure that the MSS-VM model remains a dependable tool for wind energy assessments. This comprehensive approach not only strengthens the validity of the model's predictions but also provides stakeholders with greater confidence in making informed decisions regarding wind energy projects.

## 5 Discussion

The accurate characterization of wind speed distributions is superlative for optimizing the efficiency and economic viability of wind energy projects [75,76]. This study undertakes a comprehensive evaluation of different probability distributions (PDs) to identify models that best fit wind speed data from the Gwadar region. The selection process involves rigorous statistical analysis and comparison using established goodness of fit criteria, aiming to enhance the reliability of wind resource assessments. The initial step in this study involved the consideration of 20 distinct PDs, ranging from commonly used distributions such as Gamma and Normal to more specialized models like Mixture SSVM. Each distribution was evaluated based on its ability to capture the empirical characteristics of wind speed data collected at heights of 10m and 50m in Gwadar. Based on the results from these tests, clear distinctions emerged among the PDs. At 10m height, the Mixture SSVM distribution demonstrated superior performance across multiple criteria, including AIC, BIC, AD, KS, and R^2^. This suggests its ability to effectively model the variability and distributional characteristics of wind speeds observed at lower altitudes in Gwadar. Conversely, at 50m height, the NAK-GUM distribution emerged as the most suitable model, displaying robustness in terms of AD, KS, AIC, BIC, and overall fit indices.

The complex patterns seen in wind speed data are well captured by the MSS-VM model, especially in areas like Gwadar where the wind regime can be more versatile. In contrast with traditional single distribution models, which could find it difficult to accurately depict multimodal or skewed data, by combining different distributions, the MSS-VM mixture framework enables it to provide a more complex depiction of the underlying wind speed statistics. Such flexibility is necessary to accurately simulate wind speed and variability and is necessary for accurate estimations of energy production.

The results of this research show that the MSS-VM model performs better than other distributions at several heights, especially at 10m. Its consistent performance at different altitudes indicates that it can adjust to the dynamics of wind profiles that change as height increases. This flexibility is essential for wind energy projects because the unique wind conditions at various elevations may have significant effects on turbine performance.

### 5.1 Computational efficiency of Bayesian models vs. Traditional approaches

#### 5.1.1 Bayesian models

Bayesian approaches, for instance, the Mixture SSVM (MSS-VM), offer significant versatility in simulating challenging distributions due to their ability to include prior information and modify opinions using fresh data. The Bayesian method makes use of linear models and mixtures that are better able to identify the fundamental framework of data about wind speeds. Compared to more traditional approaches. It is extremely computationally difficult to perform Bayesian inference, especially when using Markov Chain Monte Carlo (MCMC) techniques. The processor load is increased by the requirement to do computations for posterior sampling and convergence, as well as performing numerous iterations (frequently thousands). For quantifying uncertainty, Bayesian approaches offer a complete probabilistic representation of parameter uncertainty, as well as reliable interval and posterior distributions. This is especially useful for wind speed modeling, where knowing the range of potential parameter values and the uncertainty associated with their essential.

#### 5.1.2 Traditional approaches

Traditional techniques, including maximum likelihood estimation (MLE) in the context of single distribution models, may offer limited flexibility. They frequently assume a certain distribution form (e.g., Weibull, Gamma) and are unable to dynamically adjust to the complexity of the data. These techniques may have trouble processing complicated data patterns, particularly when the data does not easily fit into established distributions while being simple and requiring minimal computing resources. Traditional approaches like MLE are typically faster due to their direct optimization of likelihood functions rather than iterative sampling. These methods can be more computationally effective for simpler data structures and models with fewer parameters. In general, computing power requirements for assessing the goodness of fit for single distributions are lower than those for performing MCMC for Bayesian models, as demonstrated by Table 3, Table 4 Traditional approaches usually yield confidence intervals and point estimates, if relevant. Although helpful, this does not provide the same level of quantified uncertainty as Bayesian techniques.

### 5.2 Statistical validation

Several researchers have conducted wind speed analysis employing numerous methods [54,[77], [78], [79], [80], [81]], particularly the regional and at-site wind speed frequency analyses, that have drawn a lot of interest. The choice of an appropriate probability distribution (PD) and the estimation of its parameters are two crucial components in the assessment of severe occurrences [[82], [83], [84]]. According to studies, Weibull (WEI) distribution is a relatively commonly utilized PD for wind speed modeling [[85], [86], [87], [88]]. Additionally, the Weibel (WEI) distribution, Several PDs have been used to suit the EWS data. For instance, the Generalized Pareto (GPA) distribution was suggested to fit WS in the UK and Australia [89,90]. Correspondingly, Pandey and Sutherland [91] suggested utilizing the L-moment to estimate wind speed employing the WEI and generalized extreme value (GEV) distribution. Several PDs, including the generalized logistic (GLO), GEV, GPA, lognormal 3 (LN3), exponential (EXP), log-Pearson Type III (LP3), Pearson type 3 (P3), and WEI, for estimating wind speed [92,93]. GEV, Gamma (GAM), LP3, Gumbel (GUM), Lognormal (LN2), and WEI distributions were used in numerous studies of WSFA investigations to estimate wind speed measurements in different countries [80,[94], [95], [96]].

### 5.3 Practical implications for wind energy applications

Wind turbines produce power Without releasing greenhouse gases or other pollutants linked to standard energy sources like natural gas or coal [97]. Wind farms can damage local wildlife and ecological habitats, however, good planning and location can limit these effects [98]. Since wind farms don't need water for cooling, they use less water than thermal power plants, which eases the strain on the region's water supplies [99]. For wind energy applications in Gwadar, precisely simulating wind speed distributions is critical for projecting energy yields and optimizing turbine performance. The observed distributions offer helpful insights into the wind speed variability and peaks in the area in addition to providing a good statistical fit for the data. In practical terms, selecting an appropriate PD has significant implications for wind energy assessments. Accurate modeling of wind speed distributions informs decisions on turbine placement, operational strategies, and energy yield predictions. The findings from this study provide actionable insights into optimizing wind farm performance and enhancing energy production efficiency. Moreover, the Bayesian framework employed in MSS-VM provides a robust means of estimating design parameters critical for infrastructure planning and development.

Although the study presents insightful information, it's important to recognize any potential drawbacks, such as the assumptions that underlie each probability distribution and the distinctive characteristics of the Gwadar wind data. The outcome of this study has important ramifications for wind energy evaluation approaches. By determining the most suitable PDs for a certain height in Gwadar, Stakeholders may choose the best sites, design their turbines, and implement operational plans to optimize energy production and profitability. Technological improvements in wind energy have real-world consequences on incorporation and efficacy. Turbine design advancements that have decreased operating costs and increased efficiency include the use of larger rotor diameters and taller towers [100]. The unpredictable nature of wind power requires advancements in grid technology and energy collection instruments [101].

### 5.4 Strengths and limitations

The research uses a mixture distribution model, which fits complicated wind speed data better than single distributions. Such flexibility is essential for effectively representing the features of wind speed under various conditions. The reliability of the results is increased by using various goodness-of-fit tests (KS test, AD test, chi-square, AIC, and BIC). These tests offer a comprehensive assessment of the model's performance against multiple distribution types, guaranteeing that the best models are found for various heights (e.g., 50m and 100m). The results have important implications for the generation of wind energy, especially in terms of helping choose the right wind turbines depending on local wind conditions. This may result in more effectively produced electricity and improved financial results for wind farms.

The study limited information from Gwadar, Pakistan, which might not be typical of other areas with various geographic and meteorological characteristics. It is still necessary to test the feasibility of the model in various scenarios. Mixture models can provide difficulties in interpretation and application even while they offer superior fitting for complicated distributions. The performance of the model may be negatively impacted if the selected components do not accurately match the underlying wind speed data. Using complicated models with lots of parameters carries the danger of overfitting, which can result in inadequate generalization for fresh sets of data. Thorough validation is required to guarantee that the model is robust under various circumstances. Additionally, this study is based on secondary data collected from the Pakistan metrological department, and as such, we do not have access to detailed information regarding the measurement equipment, sampling rate, or quality assurance procedures used during data collection. This lack of specific information may limit our ability to fully assess the data quality and could introduce some uncertainty in the interpretation of the findings. We recommend that future studies collect primary data or obtain more detailed documentation to address these gaps.

## 6 Conclusion

Appropriate distribution of maximum wind speeds assessment can better understand the wind's features and possibilities for wind energy production in an observable region. It helps in the appropriate wind turbine selection as well. In the present study, the appropriate efficacy of mixture distribution is significantly influenced by the kind and number of distribution components. A mixture of SSVM distribution is suggested and utilized to estimate wind speed distribution. Based on the performance of the suggested models at Pakistan's Gwadar station, several conclusions will be drawn.

The MLE technique was employed to estimate the PDs' parameters. Among the twenty prospective PDs, the most appropriate distributions have been determined using the KS test, AD test, chi-square, AIC, BIC test, probability density function plots, probability difference plots, and Q-Q plots. The goodness-of-fit findings show that in 100m M-SSVM, KUM-N, DAG-B, Kumaraswamy, and Weibel, and at 50 m, they are M-SSVM, NAK-GAM, KUM-N, L.GA, and B were found to be the appropriate distributions for frequency assessment.

Single distribution Several model parameters increase the model's flexibility, making it better for fitting difficult wind speed distributions. When compared to other single distributions, in 10 m Kumaraswamy, and Weibel as well as at 50m log gamma, beta distribution performed better. In general, the mixture model performs better than heterogeneous mixtures and single distributions, which, using several heterogeneous mixtures, frequently perform better than single distributions.

Compared to the mixture and other single distributions, the suggested model MSSVM fit the wind speed distributions under various conditions more accurately with the smallest value of KS (0.020), AD-based indicator (0.232 m/s), Chi square-based indicator (4.182 m/s), AIC value (4724.67) and BIC-based criterion (4743.39). The suggested model will be used in the future to calculate the distribution of maximum wind speeds at unknown sites. Furthermore, the presentations of several mixture distributions, such as Kumaraswamy, beta, Weibull, log gamma, etc., will be assessed to explain the distributions of wind speeds. The MMWS estimate derived from these distributions may have significant policy implications concerning the codification of wind loads for various structural designs. By providing a more accurate representation of wind speed variability, these estimates can inform building codes and engineering standards, ensuring that structures are designed to withstand extreme wind events. This is particularly crucial for infrastructure located in areas prone to high wind speeds, as it can help mitigate potential losses due to structural failures. Policymakers can utilize this information to update existing regulations, promoting safer design practices that enhance resilience against extreme weather conditions. Furthermore, integrating these estimates into planning frameworks can facilitate the development of more robust infrastructure, ultimately reducing economic losses and enhancing public safety in the face of climate change and increasing frequency of severe weather events.

## Key massage

The study shows that the MSSVM mixture distribution model outperforms other models in fitting wind speed distributions, as indicated by superior goodness-of-fit metrics. This model accuracy at Gwadar station suggests it can effectively estimate wind speeds at new sites. Its use could enhance wind turbine selection and inform wind load regulations to better manage extreme wind conditions.

## CRediT authorship contribution statement

**Tasir Khan:** Writing – original draft, Methodology, Conceptualization. **Yejuan Wang:** Writing – review & editing, Supervision, Conceptualization.

## Data availability statement

All the authors of this manuscript have confirmed that the data supporting the study's findings are included in the article. The necessary data is both available and readily accessible.

## Consent for publication

All the authors agree to publish this paper.

## Ethical statement

All experimental procedures were authorized by the Animal Welfare and Ethics Committee at Lanzhou University (approval number LZU-201805-224).

## Declaration of competing interest

The authors declare that they have no known competing financial interests or personal relationships that could have appeared to influence the work reported in this paper.

## Figures and Tables

**Table:** 

Distributions	Abbreviations	PDF	CDF
Gamma	GAM	$f\left(x\right)=\frac{{\left(x-\gamma \right)}^{\alpha -1}}{\beta^{\alpha }\Gamma \left(\alpha \right)}\exp\;\left(-\left(x-\gamma \right)/\beta \right)$	$F\left(x\right)=\frac{\Gamma \left(x-y\right)/\beta \left(\alpha \right)}{\Gamma \left(\alpha \right)}$
Generalized Gamma	GG	$f\left(x\right)=\frac{{k\left(x-\gamma \right)}^{k\alpha -1}}{\beta^{k\alpha }\Gamma \left(\alpha \right)}\exp\;{\left(-\left(x-\gamma \right)/\beta \right)}^{k})$	$F\left(x\right)=\frac{\Gamma {\left(\left(x-y\right)/\beta \right)}^{\left(\alpha \right)}}{\Gamma \left(\alpha \right)}$
Dagum	DAG	$f\left(x\right)=\frac{{\alpha k\left(\frac{x-\gamma }{\beta }\right)}^{k\alpha -1}}{\beta {\left({1+\left(\frac{x-\gamma }{\beta }\right)}^{\alpha }\right)}^{k+1}}$	$F\left(x\right)={\left({1+\left(\frac{x-\gamma }{\beta }\right)}^{-\alpha }\right)}^{-k}$
Fatigue Life	FL	$f\left(x\right)=\frac{\sqrt{\left(x-\gamma \right)/\beta +\sqrt{\beta /\left(x-\gamma \right)}}}{2\alpha \left(x-\gamma \right)}.\emptyset \left(\frac{1}{\alpha }\left(\sqrt{\frac{x-\gamma }{\beta }}-\sqrt{\frac{\beta }{x-\gamma }}\right)\right)$	$F\left(x\right)=\Phi \left(\frac{1}{\alpha }\left(\sqrt{\frac{x-\gamma }{\beta }}-\sqrt{\frac{\beta }{x-\gamma }}\right)\right)$
Inv. Gaussian	IGA (3P)	$f\left(x\right)=\sqrt{\frac{\lambda }{{2\pi \left(x-\gamma \right)}^{3}}}\;\exp\left(-\frac{{\lambda \left(x-\gamma -\mu \right)}^{2}}{2\mu^{2}\left(x-\gamma \right)}\right)$	$F\left(x\right)=\Phi \left(\sqrt{\frac{\lambda }{x-\lambda }}\left(\frac{x-\gamma }{\mu }-1\right)\right)+\Phi \left(-\sqrt{\frac{\lambda }{x-\lambda }}\left(\frac{x-\gamma }{\mu }-1\right)\right)\;\exp\;\left(\frac{2\lambda }{\mu }\right)$
Weibull	WE (3p)	$f\left(x\right)=\frac{\sigma }{\beta }{\left(\frac{x-\gamma }{\beta }\right)}^{\alpha -1}\;\exp\;{\left(-\left(\frac{x-\gamma }{\beta }\right)\right)}^{\alpha }$	$F\left(x\right)=1-\exp\;{\left(-\left(\frac{x-\gamma }{\beta }\right)\right)}^{\alpha }$
Beta	B	$f\left(x\right)=\frac{1}{B\left(\alpha_{1},\alpha_{2}\right)}\frac{{\left(x-a\right)}^{\alpha_{1}-1}{\left(b-x\right)}^{\alpha_{2}-1}}{{\left(b-a\right)}^{\alpha_{1}+\alpha_{2}-1}}$	$F\left(x\right)=I_{z}\left(\alpha_{1},\alpha_{2}\right)$
Nakagami	NAK	$f\left(x\right)=\frac{{2m}^{m}}{\Gamma {\left(m\right)}^{\Omega m}}x^{2m-1}\;\exp\left(-\frac{m}{\Omega }x^{2}\right)$	$F\left(x\right)=\frac{\Gamma mx^{2}/\Omega^{\left(m\right)}}{\Gamma \left(m\right)}$
Johnson SU	J.SU	$f\left(x\right)=\frac{\delta }{\lambda \sqrt{2\pi }\sqrt{z^{2}+1}}\exp\left(-\frac{1}{2}{\left(\gamma +\delta \;\ln\left(z+\sqrt{z^{2}+1}\right)\right)}^{2}\right)$	$F\left(x\right)=\Phi \left(\gamma +\delta \;\ln\left(z+\sqrt{z^{2}+1}\right)\right)$
Kumaraswamy	KUM	$f\left(x\right)=\frac{\alpha_{1}\alpha_{2}z^{\alpha_{1-1}}{\left(1-z^{\alpha_{1}}\right)}^{\alpha_{2}-1}}{\left(b-a\right)}$	$F\left(x\right)={1-\left(1-z^{\alpha_{1}}\right)}^{\alpha_{2}}$ Where $z=\frac{x-a}{b-a}$
Log-Gamma	LG	$f\left(x\right)=\frac{{\left(\ln\left(x\right)\right)}^{\alpha -1}}{x\beta^{\alpha }\Gamma \left(\alpha \right)}\exp\left(-\ln\left(x\right)/\beta \right)$	$F\left(x\right)=\frac{\Gamma \;\ln\left(x\right)/\beta^{\alpha }}{\Gamma \left(\alpha \right)}$
Kumaraswamy-normal	KUM-N	$f_{KUM,N}\left(x\right)=wf_{KUM}\left(x\right)+\left(1-w\right)f_{N}\left(x\right)$	$F_{KUM,N}\left(x\right)=wF_{KUM}\left(x\right)+\left(1-w\right)F_{N}\left(x\right)$
Nakagami-Gumbel	NAK-GUM	$f_{NAK,GUM}\left(x\right)=wf_{NAK}\left(x\right)+\left(1-w\right)f_{GUM}\left(x\right)$	$F_{NAK.GUM}\left(x\right)=wF_{NAK}\left(x\right)+\left(1-w\right)F_{GUM}\left(x\right)$
Log Pearson-generalize extreme value	LP-GEV	$f_{LP,GEV}\left(x\right)=wf_{LP}\left(x\right)+\left(1-w\right)f_{GEV}\left(x\right)$	$F_{LP,GEV}\left(x\right)=wF_{LP}\left(x\right)+\left(1-w\right)F_{GEV}\left(x\right)$
Dagum-Beta	DAG-B	$f_{DAG,B}\left(x\right)=wf_{DAG}\left(x\right)+\left(1-w\right)f_{B}\left(x\right)$	$F_{DAG,B}\left(x\right)=wF_{DAG}\left(x\right)+\left(1-w\right)F_{B}\left(x\right)$
Log normal-log Pearson	LN-LP	$f_{\mathrm{LN},LP}\left(x\right)=wf_{\mathrm{LN}}\left(x\right)+\left(1-w\right)f_{LP}\left(x\right)$	$F_{\mathrm{LN},LP}\left(x\right)=wF_{\mathrm{LN}}\left(x\right)+\left(1-w\right)F_{LP}\left(x\right)$
Log logistics- inverse gaussian	LL-IG	$f_{LL,IG}\left(x\right)=wf_{LL}\left(x\right)+\left(1-w\right)f_{IG}\left(x\right)$	$F_{LL,IG}\left(x\right)=wF_{LL}\left(x\right)+\left(1-w\right)F_{IG}\left(x\right)$
Normal-Gamma	N-GAM	$f_{N,GAM}\left(x\right)=wf_{N}\left(x\right)+\left(1-w\right)f_{GAM}\left(x\right)$	$F_{N,GAM}\left(x\right)=wF_{N}\left(x\right)+\left(1-w\right)F_{GAM}\left(x\right)$

## Appendix B

Proof of Sine- Skewed von Mises Distribution

(1) $$f_{SSVM}\left(\theta ;\mu ,\tau ,\lambda \right)=\frac{e\text{xp}\;(k\;\cos\;\left(\theta_{1}\right)}{2\pi I_{0}\left(k\right)}(1+\lambda \;\sin\;\left(\theta_{1}\right)$$

If the random variable θ has the pdf as given in (1), then

$$E\left(\theta \right)=\int_{-\pi }^{\pi }\frac{e\text{xp}\;(k\;\cos\;\left(\theta_{1}\right)}{2\pi I_{0}\left(\theta_{1}\right)}(1+\lambda \;\sin\;\left(\theta_{1}\right)d\theta_{1}$$

$$=\int_{-\pi }^{\pi }\frac{\theta_{1}e\text{xp}\;(k\;\cos\;\left(\theta_{1}\right)}{2\pi I_{0}\left(k\right)}d\theta_{1}+\lambda \int_{-\pi }^{\pi }\frac{\theta_{1}e\text{xp}\;(k\;\cos\;\left(\theta_{1}\right)}{2\pi I_{0}\left(k\right)}\sin\;\theta_{1}d\theta_{1}$$

$$=\int_{-\pi }^{\pi }\frac{\theta_{1}}{2\pi I_{0}\left(k\right)}\left(I_{0}\left(k\right)+2\sum_{j=1}^{\infty }I_{j}\left(k\right)cosj\theta_{1}\right)d\theta_{1}+\frac{\lambda }{k}{\left({-\theta }_{1}\frac{e^{kcos\theta_{1}}}{2\pi I_{0}\left(k\right)}\right)|}_{-\pi }^{\pi }++\frac{\lambda }{k}\int_{-\pi }^{\pi }\frac{e^{kcos\theta_{1}}}{2\pi I_{0}\left(k\right)}d\theta_{1}$$

$$=\frac{\lambda }{2\pi I_{0}\left(k\right)}\left(-\frac{2\pi }{k}e^{-k}+\frac{2\pi }{k}I_{0}\left(k\right)\right)$$

$$=-\frac{1}{k}\frac{\lambda }{I_{0}\left(k\right)}\left(e^{-k}-I_{0}\left(k\right)\right)$$

$$E\left(\theta \right)=\frac{\lambda }{k}\left(1-\frac{e^{-k}}{I_{0}\left(k\right)}\right)$$

We define the $p^{th}$ circular moment $\varphi_{p}$ of random variable θ with pdf f(θ), with π≤θ≤π as

$$\varphi_{p}=\int_{-\pi }^{\pi }e^{ip\theta }\;f\left(\theta \right)d\theta$$

### Characterizations

We shall now derive the two characterizations of the skew von Mises distribution. We will need the following two lemmas for the characterization of the skew von Mises distribution by truncated moment.

### Assumption A

Let θ be a continuous random variable with cdf $F\left(\theta_{1}\right)$ and pdf $f\left(\theta_{1}\right)$. Assume that E(θ) exists and f(θ) is differentiable. We assume further that $\alpha =\sup\left\{\theta_{1}/F\left(\theta_{1}\right)\;a\right\}$ and $\beta =f\left\{\theta_{1}/F\left(\theta_{1}\right)<1\right\}$. Lemma 1 .

If $\left(\theta /\theta \leq \theta_{1}\right)=g\left(\theta_{1}\right)\frac{f\left(\theta_{1}\right)}{F\left(\theta_{1}\right)}$ , where $g\left(\theta_{1}\right)$ is a continuous differentiable function in α≤θ≤β then

$$f\left(\theta \right)=cexp\left(\int \frac{\theta -g^{\prime }\left(\theta \right)d\theta }{g\left(\theta \right)}\right)\text{,}$$

Where c is determined by the condition $\int_{\alpha }^{\beta }f\left(\theta_{1}\right)d\theta =1$. proof .

We know from elementary theory that

$$E\left(\theta /\theta \leq \theta_{1}\right)=\frac{\int_{\alpha }^{\theta_{1}}uf\left(u\right)}{F\left(\theta_{1}\right)}$$

$$g\left(\theta_{1}\right)=\frac{\int_{\alpha }^{\theta_{1}}uf\left(u\right)}{f\left(\theta_{1}\right)}$$

$$\int_{\alpha }^{\theta_{1}}uf\left(u\right)du=f\left(\theta_{1}\right)g\left(\theta_{1}\right)$$

Differentiate to $\theta_{1}$ both sides

$$\theta_{1}f\left(\theta_{1}\right)=f\left(\theta_{1}\right)g^{\prime }\left(\theta_{1}\right)+f^{\prime }\left(\theta_{1}\right)g\left(\theta_{1}\right)$$

$$\frac{f^{\prime }\left(\theta_{1}\right)}{f\left(\theta_{1}\right)}=\frac{\theta_{1}-g^{\prime }\left(\theta_{1}\right)}{g\left(\theta_{1}\right)}$$

Intergrade both sides

$$f\left(\theta \right)=c.\exp\left(\int \frac{\theta -g^{\prime }\left(\theta \right)}{g\left(\theta \right)}d\theta \right)\text{,}$$

where c is determined by the condition $\int_{\alpha }^{\beta }f\left(\theta_{1}\right)d\theta =1$. Lemma 1 *Under the Assumption A*, *if*$E\left(\theta /\theta \leq \theta_{1}\right)=h\left(\theta_{1}\right)\frac{f\left(\theta_{1}\right)}{1-F\left(\theta_{1}\right)}$, *where*h(θ)*is a*

$$f\left(\theta \right)=cexp\left(-\int \frac{\theta -h^{\prime }\left(\theta \right)}{h\left(\theta \right)}d\theta \right)\text{,}$$

where c is determined by the condition $\int_{\alpha }^{\beta }f\left(\theta_{1}\right)d\theta =1$. Proof we know that

$$E\left(\theta /\theta \leq \theta_{1}\right)=\frac{\int_{\theta_{1}}^{\beta }uf\left(u\right)}{1-F\left(\theta_{1}\right)}$$

Hence, equating the RHS of both sides, we have,

$$h\left(\theta_{1}\right)=\frac{\int_{\theta_{1}}^{\beta }uf\left(u\right)du}{f\left(\theta_{1}\right)}\text{,}$$

$$h\left(\theta_{1}\right)h\left(\theta_{1}\right)=\int_{\theta_{1}}^{\beta }uf\left(u\right)du$$

Differentiate with respect to $\theta_{1}$ both sides

$$f\left(\theta_{1}\right)h^{\prime }\left(\theta_{1}\right)=f^{\prime }\left(\theta_{1}\right)h\left(\theta_{1}\right)=-\theta_{1}f\left(\theta_{1}\right)$$

Re-arrange

$$\frac{f^{\prime }\left(\theta_{1}\right)}{f\left(\theta_{1}\right)}=\frac{\theta_{1}-h^{\prime }\left(\theta_{1}\right)}{h\left(\theta_{1}\right)}$$

integrating both sides,

$$f\left(\theta \right)=cexp\left(-\int \frac{\theta -h^{\prime }\left(\theta \right)}{h\left(\theta \right)}d\theta \right)\text{.}$$

This completes the proof. Theorem 1 .

Suppose that the random variable θ satisfies the conditions given in Assumption A, with pdf f(θ), cdf F(θ), α=−π and β=π. Then $E\left(\theta /\theta \leq \theta_{1}\right)=f\left(\theta_{1}\right)\tau \left(\theta_{1}\right)$,

$$\tau \left(\theta_{1}\right)=\frac{f\left(\theta_{1}\right)}{F\left(\theta_{1}\right)}\text{,}$$

$$g\left(\theta_{1}\right)=\frac{2\pi I_{0}\left(k\right)p\left(\theta_{1}\right)}{F\left(\theta_{1}\right)e^{kcos\theta_{1}}\left(1+\lambda \;\sin\;\theta_{1}\right)}$$

and

$$\tau \left(\theta_{1}\right)=\frac{\theta_{1}^{2}-\pi^{2}}{4\pi }+\frac{1}{\pi I_{0}\left(k\right)}\sum_{j=1}^{\infty }I_{j}\left(k\right)\left\{\frac{1}{j}\theta_{1}\;sinj\theta_{1}+\frac{1}{j^{2}}cosj\theta_{1}-\frac{{\left(-1\right)}^{j}}{j^{2}}\right\}-\frac{\lambda }{2\pi I_{0}\left(k\right)}\left(\pi e^{-k}+\theta_{1}e^{kcos\theta_{1}}\right)+\lambda \frac{\theta_{1}+\pi }{2k\pi }+\frac{1}{\pi I_{0}\left(k\right)}\sum_{j=1}^{\infty }I_{j}\left(k\right)\frac{1}{j}sinj\theta_{1}$$

If and only if θ has the skew von Mises distribution with pdf $f_{\theta }\left(\theta_{1}\right)$ as

$$f_{\theta }\left(\theta_{1}\right)=\frac{e\text{xp}\;(k\;\cos\;\left(\theta_{1}\right)}{2\pi I_{0}\left(k\right)}(1+\lambda \;\sin\;\left(\theta_{1}\right)\text{,}$$

$-\pi \leq \theta_{1}\leq \pi ,-1\leq \lambda \leq 1$ and *k* is any real number. Proof .

$$f\left(\theta \right)=\frac{\exp\;\left(\text{kcos}\left(\theta \right)\right)}{2\pi I_{0}\left(k\right)}\left(1+\lambda \;\sin\;\theta \right)\text{,}$$

Then

$$g\left(\theta_{1}\right)f\left(\theta_{1}\right)=\int_{-\pi }^{\theta_{1}}\frac{e^{\text{kcos}\left(\theta \right)}}{2\pi I_{0}\left(k\right)}\left(1+\lambda \;\sin\;\theta \right)d\theta$$

$$=\int_{-\pi }^{\theta_{1}}\frac{e^{\text{kcos}\left(\theta \right)}}{2\pi I_{0}\left(k\right)}\;d\theta +\int_{-\pi }^{\theta_{1}}\frac{\theta e^{\text{kcos}\left(\theta \right)}}{2\pi I_{0}\left(k\right)}\;\sin\;\theta d\theta$$

$$=\int_{-\pi }^{\theta_{1}}\frac{\theta }{2\pi I_{0}\left(k\right)}\left\{I_{0}\left(k\right)+2\sum_{j=1}^{\infty }I_{j}\left(k\right)cosj\theta d\theta \right\}+{\lambda \left(-\theta \frac{e^{kcos\theta }}{2\pi I_{0}\left(k\right)}\right)|}_{-\pi }^{\theta_{1}}+\lambda \int_{-\pi }^{\theta_{1}}\frac{e^{\text{kcos}\left(\theta \right)}}{2\pi I_{0}\left(k\right)}\;d\theta$$

$$=\frac{\theta_{1}^{2}-\pi^{2}}{4\pi }+\frac{\theta }{2\pi I_{0}\left(k\right)}\sum_{j=1}^{\infty }I_{j}\left(k\right)\left\{\frac{1}{j}\theta_{1}\;\sin\;\theta_{1}+\frac{1}{j^{2}}cosj\theta_{1}-\frac{{\left(-1\right)}^{j}}{j^{2}}\right\}-\frac{\lambda }{2\pi I_{0}\left(k\right)}\left({\pi e}^{-k}+\theta_{1}e^{\cos\;\theta_{1}}\right)+\lambda \frac{\theta_{1}+\pi }{2k\pi }+\frac{1}{\pi I_{0}\left(k\right)}\sum_{j=1}^{\infty }I_{i}\left(k\right)\frac{1}{j}sinj\theta_{1}$$

$$=p\left(\theta_{1}\right)\text{,}$$

Thus,

$$g\left(\theta_{1}\right)=\frac{2\pi I_{0}\left(k\right)p\left(\theta_{1}\right)}{e^{kcos\theta_{1}}\left(1+\lambda \;\sin\;\theta_{1}\right)}$$

Suppose that

$$g\left(\theta_{1}\right)=\frac{2\pi I_{0}\left(k\right)p\left(\theta_{1}\right)}{e^{kcos\theta_{1}}\left(1+\lambda \;\sin\;\theta_{1}\right)}$$

Then

$$g^{\prime }\left(\theta_{1}\right)=\theta_{1}-\frac{2\pi I_{0}\left(k\right)p\left(\theta_{1}\right)}{e^{kcos\theta_{1}}\left(1+\lambda \;\sin\;\theta_{1}\right)}\left\{-k\;\sin\;\theta_{1}+\frac{\lambda \;\cos\;\theta_{1}}{1+\lambda \;\sin\;\theta_{1}}\right\}$$

$$=\theta_{1}-g\left(\theta_{1}\right)\left\{-k\;\sin\;\theta_{1}+\frac{\lambda \;\cos\;\theta_{1}}{1+\lambda \;\sin\;\theta_{1}}\right\}$$

Hence

$$\frac{\theta_{1}-g^{\prime }\left(\theta_{1}\right)}{g\left(\theta_{1}\right)}=-k\;\sin\;\theta_{1}+\frac{\lambda \;\cos\;\theta_{1}}{1+\lambda \;\sin\;\theta_{1}}$$

By Lemma 1

$$\frac{f^{\prime }\left(\theta_{1}\right)}{g\left(\theta_{1}\right)}=-k\;\sin\;\theta_{1}+\frac{\lambda \;\cos\;\theta_{1}}{1+\lambda \;\sin\;\theta_{1}}$$

Integrating the above equation with respect to $\theta_{1}$.

$$f\left(\theta \right)={ce}^{kcos\theta }\left\{1+\lambda \;\sin\;\theta \right\}$$

Using the condition $\int_{-\pi }^{\pi }f\left(\theta \right)d\theta =1\text{,}$ we obtain

$$f\left(\theta \right)=\frac{e^{kcos\theta }}{2\pi I_{0}\left(k\right)}\left\{1+\lambda \;\sin\;\theta \right\}$$

Theorem 2 .

Suppose that the random variable θ satisfies the conditions given in Assumption A, with α=−π and β=π. Then

$$E\left(\theta /\theta \leq \theta_{1}\right)=\frac{2\pi I_{0}\left(k\right)h\left(\theta_{1}\right)}{e^{kcos\theta_{1}}\left(1+\lambda \;\sin\;\theta_{1}\right)}r\left(\theta_{1}\right)$$

$$r\left(\theta_{1}\right)=\frac{f\left(\theta_{1}\right)}{1-F\left(\theta_{1}\right)}\text{,}$$

and

$$h\left(\theta_{1}\right)=\frac{2\pi I_{0}\left(k\right)\left\{E\left(\theta \right)-p\left(\theta_{1}\right)\right\}}{e^{kcos\theta_{1}}\left(1+\lambda \;\sin\;\theta_{1}\right)}$$

$$f\left(\theta \right)=\frac{e^{kcos\theta }}{2\pi I_{0}\left(k\right)}\left\{1+\lambda \;\sin\;\theta \right\}\text{,}$$

Proof .

If the pdf of the random variable θ is $f\left(\theta \right)=\frac{e^{kcos\theta }}{2\pi I_{0}\left(k\right)}\left\{1+\lambda \;\sin\;\theta \right\}\text{,}$ then

$$f\left(\theta_{1}\right)h\left(\theta_{1}\right)=\int_{\theta_{1}}^{\pi }\theta f\left(\theta \right)d\theta =E\left(\theta \right)-p\left(\theta_{1}\right)$$

Thus

$$h\left(\theta_{1}\right)=\frac{2\pi I_{0}\left(k\right)\left\{E\left(\theta \right)-p\left(\theta_{1}\right)\right\}}{e^{kcos\theta_{1}}\left(1+\lambda \;\sin\;\theta_{1}\right)}$$

Suppose that

$$h\left(\theta_{1}\right)=\frac{2\pi I_{0}\left(k\right)\left\{E\left(\theta \right)-p\left(\theta_{1}\right)\right\}}{e^{kcos\theta_{1}}\left(1+\lambda \;\sin\;\theta_{1}\right)}\text{,}$$

then

$$h^{\prime }\left(\theta_{1}\right)==\theta_{1}-\frac{2\pi I_{0}\left(k\right)\left\{E\left(\theta \right)-p\left(\theta_{1}\right)\right\}}{e^{kcos\theta_{1}}\left(1+\lambda \;\sin\;\theta_{1}\right)}\left(-ksin\theta_{1}+\frac{kcos\theta_{1}}{1+\lambda \;\sin\;\theta_{1}}\right)$$

$$==\theta_{1}-h\left(\theta_{1}\right)\left(-ksin\theta_{1}+\frac{kcos\theta_{1}}{1+\lambda \;\sin\;\theta_{1}}\right)$$

$$-\frac{{\theta_{1}+h}^{\prime }\left(\theta_{1}\right)}{h\left(\theta_{1}\right)}=-ksin\theta_{1}+\frac{kcos\theta_{1}}{1+\lambda \;\sin\;\theta_{1}}\text{,}$$

Thus, by Lemma 2

$$\frac{h^{\prime }\left(\theta_{1}\right)}{h\left(\theta_{1}\right)}=-ksin\theta_{1}+\frac{kcos\theta_{1}}{1+\lambda \;\sin\;\theta_{1}}$$

On integrating the above equation with respect to $\theta_{1}$, we obtain $f\left(\theta \right)={ce}^{kcos\theta }\left(1+\lambda \;\sin\;\theta \right)$.

Using the condition $\int_{-\pi }^{\pi }f\left(\theta \right)d\theta =1\text{,}$ we obtain

$$f\left(\theta \right)=\frac{e^{kcos\theta }}{2\pi I_{0}\left(k\right)}\left\{1+\lambda \;\sin\;\theta \right\}$$
